# The Depression Anxiety Stress Scale 8-Items Expresses Robust Psychometric Properties as an Ideal Shorter Version of the Depression Anxiety Stress Scale 21 Among Healthy Respondents From Three Continents

**DOI:** 10.3389/fpsyg.2022.799769

**Published:** 2022-03-24

**Authors:** Amira Mohammed Ali, Hiroaki Hori, Yoshiharu Kim, Hiroshi Kunugi

**Affiliations:** ^1^Department of Behavioral Medicine, National Institute of Mental Health, National Center of Neurology and Psychiatry, Tokyo, Japan; ^2^Department of Psychiatric Nursing and Mental Health, Faculty of Nursing, Alexandria University, Alexandria, Egypt; ^3^Department of Psychiatry, Teikyo University School of Medicine, Tokyo, Japan; ^4^Department of Mental Disorder Research, National Institute of Neuroscience, National Center of Neurology and Psychiatry, Tokyo, Japan

**Keywords:** psychological distress/stress/anxiety/depression, short form of the depression anxiety stress scale 21 (DASS-21)/depression anxiety stress scale 8-items, factorial structure/psychometric properties/predictive validity/convergent validity/validation/measurement invariance, healthy individuals, students, coronavirus disease 2019/COVID-19, cultur*/collectiv*/individual*, countr*/Australia/United States/Ghana

## Abstract

To examine the cultural limitations and implications in the applicability of the Depression Anxiety Stress Scale 8-items (DASS-8)—a shortened version of the DASS-21 recently introduced in an Arab sample—this study evaluated its psychometric properties, including measurement invariance, among healthy subjects from the United States, Australia, and Ghana. Confirmatory factor analysis revealed good fit of the DASS-8 relative to a 12-item version (DASS-12). Both the DASS-8 and the DASS-12 were invariant at all levels across genders, employment status, and students vs. non-students. The DASS-8/DASS-12 also expressed invariance at the configural and metric levels across all countries, albeit scalar invariance was not maintained due to misspecification of the factor loadings in the Ghanian sample. Mann–Whitney U test revealed significantly lower levels of mental symptomatology on the DASS measures among Ghanian students than in English-speaking respondents (both students and non-students). The DASS-8 expressed excellent internal consistency (coefficient alpha = 0.89), good convergent validity—noted by high values of item-total correlations (*r* = 0.87 to 0.88), good predictive validity—indicated by significantly strong correlation with the DASS-21 and its subscales (*r* = 0.95 to 0.80), and adequate discriminant validity—indicated by heterotrait–monotrait ratio of correlations <0.85. The DASS-8 correlated with the Internet Gaming Disorder-9, the Adult attention-deficit/hyperactivity disorder Self-Report Scale, and the Individualism and Collectivism Scale/Culture Orientation Scale at the same level as the DASS-21 and the DASS-12, denoting its adequate criterion validity. The DASS-8 can be used as a brief alternative to the DASS-21 to screen for mental symptomatology in English-speaking and African cultures. However, the same scores on the DASS-8 and the DASS-12 may not always indicate the same level of symptom severity in subjects from different countries. Further inter-cultural evaluations of the DASS-8 are needed.

## Introduction

Depression and anxiety disorders are commonly occurring conditions that are frequently comorbid with several mental and physical disorders (Lovibond and Lovibond, 1995; Vignola and Tucci, 2014). They frequently result from genetic, nutritional, psychosocial (e.g., childhood trauma), and systemic inflammatory and oxidative stress factors (e.g., COVID-19 infection; Ali et al., 2021e). They have several symptoms in common. Therefore, enormous research efforts have been invested in developing and testing measures capable of depicting the distinct features of depression and anxiety (Lovibond and Lovibond, 1995; Osman et al., 2012). Measures commonly used to assess depression or anxiety alone, such as the Beck Depression Inventory (BDI) and the Beck Anxiety Inventory (BAI), fail to discriminate between depression and other affective states because they comprise items that measure non-specific symptoms such as weight loss, insomnia, somatic preoccupation, and irritability (Lovibond and Lovibond, 1995).

The Depression, Anxiety, and Stress Scale-21 (DASS-21), a short form of the DASS-42, was developed based on the tripartite model of psychopathology (Vignola and Tucci, 2014). This model assumes that depression, anxiety, and stress comprise a general construct of overall distress while retaining distinct features that characterize the three constructs. In this respect, the DASS-21 was designed to detect the distinct features of depression, anxiety, and stress (Lovibond and Lovibond, 1995; Osman et al., 2012; Corcoran and McNulty, 2018). Depression is reflected by symptoms of hopelessness, dysphoria, anhedonia, devaluation of life, and self-deprecation. Anxiety is reflected by symptoms of physiological arousal, skeletal muscle tightening, and subjective experience of anxious affect. Stress is described by symptoms that cooccur in both conditions representing stress (e.g., irritability, nervousness, and agitation; Caetano et al., 2017; Corcoran and McNulty, 2018; Mazza et al., 2020). Because the DASS-21 is simple and measures three constructs, it has been translated into 45 languages (Zanon et al., 2020) and has been frequently used as a measure of depression and anxiety psychopathological symptoms both in research and first-line clinical services (Lovibond and Lovibond, 1995; Ali and Green, 2019).

Consistent with clinical reports on the comorbidity of mood and anxiety disorders (Janiri et al., 2020; ter Meulen et al., 2021), investigations of the structure of the DASS-21 in numerous samples of university students in the United States (US) revealed that a general distress factor accounts for the majority of variance of both the scale items and its total score. Therefore, the DASS-21 can be also used as a measure of psychological distress (Osman et al., 2012; Corcoran and McNulty, 2018). Despite extensive literature, the concept of psychological distress is largely diversified: referring mainly to mental symptoms of depression, anxiety, in addition to personality traits and numerous psychological (e.g., burnout), somatic, and behavioral problems (Dyrbye et al., 2006; Drapeau et al., 2012). Kessler Psychological Distress Scale is based on the conceptualization of psychological distress as non-specific negative emotions of combined feelings of anxiety and depression, which are closely associated with mental disorders (Zhang et al., 2018). In accordance, this study adopts the definition of psychological distress proposed by Drapeau et al. (2012): “a state of emotional suffering characterized by symptoms of depression and anxiety.” During the current COVID-19 pandemic, scores of standalone measures of depression and anxiety, such as Patient Health Questionnaire and 7-item Generalized Anxiety Disorder, have been combined to identify the level of psychological distress associated with the pandemic in China (Zhang et al., 2020). Alternatively, the DASS-21 as a single measure, which reflects on symptoms of depression, anxiety, and stress has been one of the most widely used scales to assess psychological distress associated with living within the context of the COVID-19 pandemic in different parts of the world, including China, Italy, Spain, Saudi Arabia, etc. (Cooke et al., 2020; Mazza et al., 2020; Vahedian-Azimi et al., 2020; Guerrini Usubini et al., 2021; Masuyama et al., 2021; Ali et al., 2021a).

Despite the fact that the DASS-21 demonstrates concurrent validity with parallel measures of emotional negativity (e.g., BDI, BAI, and the State–Trait Anxiety Inventory Trait; Le et al., 2017), validation findings on its factorial structure are inconsistent across different cultures (Zanon et al., 2020). For instance, a four-factor structure was obtained in Vietnamese adolescents (Le et al., 2017) whereas a two-factor structure was obtained in Brazilian adolescents (Silva et al., 2016), and a three-factor structure was replicated in Brazilian adults (Vignola and Tucci, 2014). Investigations involving other adult participants exhibit similar variations (Ali et al., 2021a). A bifactor structure describes the best fit for the data in an investigation of dimensionality of the DASS-21 in a large sample of college students (*N* = 2,580) from Brazil, Canada, Hong Kong, Romania, Taiwan, Turkey, United Arab Emirates, and the US (Zanon et al., 2020). However, measurement invariance revealed configural invariance of the bifactor structure across countries, denoting that this model does not fit in some subsamples. As a result, the authors recommended using the scale as an overall measure of distress rather than a measure of depression, anxiety, and stress (Zanon et al., 2020). In a sample of healthy individuals (*N* = 717), combining items of the BDI and BAI revealed a factor structure virtually identical to that formerly described by Beck for a sample of patients diagnosed with depression and anxiety, suggesting the view that depression and anxiety as clinical states can be considered as more severe expressions of the same states in normal individuals (Lovibond and Lovibond, 1995). Many studies also report cross-loading of items of the DASS-21 on two factors or more (Mahmoud and Hall, 2010; Oei et al., 2013; Tonsing, 2014; Wang et al., 2016; Jun et al., 2018; Teo et al., 2019), denoting that many items on the DASS-21 do not differentiate among the three constructs that the scale is intended to measure. Instead, they reflect a shared characteristic among these constructs (Caetano et al., 2017; Ali and Green, 2019). In accordance, the DASS-21 is reported to differentiate individuals with psychiatric disorders from healthy individuals. Yet, in most instances, it does not differentiate patients with depression from those with anxiety disorders (Gloster et al., 2008; Yıldırım et al., 2018). In this respect, the DASS-21 can provide a dimensional rather than a categorical conception of psychopathological disorders. Therefore, it may not be used make a final clinical diagnosis (Lovibond and Lovibond, 1995; Caetano et al., 2017).

Increasing case detection in routine primary health care settings and management of psychopathologies by non-specialized workers are among the top research priorities identified by the Grand Challenges in Global Mental Health initiative, which is launched by the National Institute of Mental Health, entailing a great need for globally valid and reliable brief psychiatric screening instruments (Sweetland et al., 2014). In this context, a two-phase procedure to screen for depression and anxiety may reduce the time and cost of screening given lack of resources allocated for intensive, clinician-administered differential diagnostic procedures, especially in large groups (Shrout and Yager, 1989). Initial screening with a shorter version of the DASS-21 may facilitate the selection of individuals likely to have depression or anxiety while eliminating individuals unlikely to endorse psychopathological disorders. Then, a detailed diagnostic workup would confirm the diagnosis in the selected group (Shrout and Yager, 1989).

From a practical point of view, the main constructs measured by the DASS-21 can be reliably depicted by a valid subset of items on the scale. In two studies using confirmatory factor analysis (CFA) and item bifactor analysis of the DASS-21 in two university student samples, Osman et al. have previously indicated that 13 items of the DASS-21 had significant loadings on their domain-specific factors. Four of these items had loadings below 0.5 indicating moderate association with the corresponding factors (Osman et al., 2012). Because the reliability of the symptom scale score can be a direct function of the number of items, most scales are usually designed to include 20 items or more in order to express high reliability. However, not all items considerably contribute to the true reliability of a scale (Shrout and Yager, 1989). In a number of studies, many items have been eliminated from the DASS-21 without altering its reliability (Ali and Green, 2019; Lee et al., 2019; Ali et al., 2021a). Indeed, based on Osman’s findings, Lee et al. developed a 12-item DASS (Lee et al., 2019). However, that DASS-12 left out some items with moderate and high loadings based on Osman’s analysis (e.g., items 8, 16, and 21). Instead, it contained other items, which are reported to have low loadings on the corresponding factors in Osman’s study such as item 11 and 18 (Osman et al., 2012). These items are also reported to have very low loadings or did not load on any factor in other previous studies (Sinclair et al., 2012; Ali and Green, 2019). The authors did not provide a rationale for including these items or for leaving out items indicated to have high loadings in Osman’s analysis (Lee et al., 2019).

In a recent investigation, the two models suggested by Osman et al. (13 items and 9 items) expressed suboptimal fit, and the DASS-21 has been reduced to an 8-item version (DASS-8) based on a revision of item loadings, item-total correlations, and the most prevalent symptoms of depression, anxiety, and stress that are reported in the literature. Psychometric comparisons revealed superior properties of the DASS-8 relative to the DASS-21 and the DASS-12 in psychiatric patients and healthy individuals (Ali et al., 2021a). Same as the DASS-21, both the DASS-8 and the DASS-12 could differentiate between healthy and psychiatric participants but lacked the capacity to differentiate between depression and anxiety as diagnoses (Lee et al., 2019; Ali et al., 2021a).

The DASS-8 was examined only in Arab samples from an Asian country in the Middle East region. Asian patients tend to report somatic symptoms (e.g., dizziness) rather than emotional symptoms. However, they admit the presence of emotional symptoms with further probing (Office of the Surgeon General (US) et al., 2001). Investigations of emotional regulation (processes that shape the type of experienced emotions one has, when they are experienced, and how they are expressed) across participants from the US and four Arab countries from the Middle East show that Arabs exhibit higher levels of expressive suppression, rumination, other-blame, and negative affectivity as well as lower scores on self-efficacy in expressing/managing positive emotions than American counterparts (Megreya et al., 2018). Culture and social contexts are major determinants of how individuals conceptualize, express, and cope with emotional illnesses. Evidence shows that individuals in different cultures selectively express their symptoms *via* culturally acceptable ways (Office of the Surgeon General (US) et al., 2001; Sweetland et al., 2014; Megreya et al., 2018), which may affect the way they respond to a symptom scale. In fact, the literature reports vivid variations in the psychometrics of the DASS-21 in populations from different countries (Scholten et al., 2017; Bibi et al., 2020; Zanon et al., 2020). This can be attributed to the fact that psychopathologies addressed by the DASS-21 such as depression, which expresses a variable global prevalence (3–19%), are mostly affected by cultural and social factors such as poverty, violence, life stress, as well as numerous factors associated with ethnicity or country of origin (Office of the Surgeon General (US) et al., 2001). This notion is supported by molecular biology and genetic studies, which report lower heritability of depression comparable with schizophrenia and bipolar disorder, which have a stable global prevalence of 1% and 0.4–1.5%, respectively (Office of the Surgeon General (US) et al., 2001).

Expressive suppression is higher among individuals from collectivistic cultures (e.g., in the Middle East and sub-Saharan Africa), which emphasize interdependence and in-grou*p* values than those from individualistic cultures (e.g., the US), which emphasize individual values and independence (Sweetland et al., 2014; Megreya et al., 2018). Therefore, the current study aimed to evaluate the psychometric properties of the DASS-8 in an international sample from three countries with different cultural orientations. The objectives of the present study were to: (1) examine the factor structure of the DASS-8 and the DASS-12 using CFA; (2) evaluate measurement invariance of the DASS-8 vs. the DASS-12 across various characteristics of the participants; (3) attain estimates of the internal consistency of the DASS-8; (4) examine criterion validity of the DASS-8 vs. that of the parent scale and the DASS-12, and (5) examine the discriminant validity of the DASS-8/DASS-12. Based on a single available investigation of the DASS-8 (Ali et al., 2021a), we hypothesized that the DASS-8 would express better fit than the DASS-12 among participants from different cultural backgrounds. Nonetheless, based on reports of variance of different structures of the DASS-21 among samples from different countries (Scholten et al., 2017; Bibi et al., 2020; Zanon et al., 2020), we hypothesized that the DASS-8/DASS-12 may be variant across English-speaking and Ghanian participants. Because emotional negativity is associated with dysfunctional behaviors and psychopathology (Dyrbye et al., 2006; Drapeau et al., 2012; Zhang et al., 2018), we hypothesized that all versions of the DASS would positively correlate with internet gaming disorder and symptoms of attention-deficit hyperactivity. Since cognitive emotional regulation and regulatory emotion self-efficacy tend to be highly expressed in individualistic cultures (Megreya et al., 2018), we expected that participants with higher scores on individualism scales would express lower levels of mental symptoms contrary to participants with higher scores on collectivism scales.

## Materials and Methods

### Study Design, Participants, and Procedure

This cross-sectional study is a secondary analysis based on two publicly accessible data sets that comprise two convenient samples collected *via* anonymous online surveys. The first sample (*N* = 1,032) is in the public domain (Burleigh, 2019). It was collected *via* SurveyGizmo. Participation was eligible for individuals aged 18 years or older, who had previous experience with gaming applications and were residing in some English-speaking countries: Australia (*N* = 738, 71.5%), the US (*N* = 222, 21.5%), the United Kinhdome (*N* = 7, 0.7%), New Zealand (*N* = 14, 1.4%), or other locations (*N* = 51, 4.9%). Around half the participants were females (*N* = 529, 51.3%), and most of the participants were employed (*N* = 743, 71.9%). As for their educational level, (*N* = 56, 5.4%) had elementary school, (*N* = 366, 35.5%) had high school, (*N* = 330, 32.0%) had a university degree while (*N* = 279, 27.0%) had an intermediate degree between high school and university; (*N* = 345, 33.4%) were currently students. For 964 participants who reported their age, the average age was 25.7 ± 7.6 years, range = 18–72 years. Participation was voluntary, and the ethics committee of Cairnmillar Institute approved the data collection procedure (Andreetta et al., 2020).

The second sample (*N* = 392) is shared under the terms of creative common license (CC BY 4.0; Wireko-Gyebi, 2020). It comprised Ghanaian university students who were mostly single (*N* = 358, 91.3%) females (*N* = 289, 73.7%). As for their age, 13.8, 62.5, and 23.7% of the participants were in the age categories of 16–20 years, 21–25 years, and more than 25 years, respectively. The data set is affiliated with the University of Energy and Natural Resources, with less information available on the methodologies used.

### Study Instruments

Few sociodemographic characteristics were collected from the Ghanian participants such as age (a categorical variable—described above), gender, and marital status. The sociodemographic characteristics collected from the English-speaking participants included age, gender, level of education, employment status (employed vs. unemployed), etc. The questionnaire addressed to the Ghanian and English-speaking participants comprised the DASS-21, a measure of mental symptoms of depression, anxiety, and stress. It comprises 21 items in three subscales; each subscale comprises seven items. Items are rated on a 4-point scale ranging from (0 = did not apply to me at all) to (3 = applied to me very much or most of the time). The total score of the DASS-21 ranges between 0 and 63 (Lovibond and Lovibond, 1995). The reliability of the DASS-21 in the English-speaking and Ghanaian participants was excellent (coefficient alpha = 0.93 and 0.95, respectively).

The Adult attention-deficit/hyperactivity disorder (ADHD) Self-Report Scale (ASRS) is a brief measure of current symptoms of ADHD. It comprises 18 items, in three subscales (inattention, hyperactivity, and impulsivity). Symptom severity is rated on a 5-point Likert scale (0 = never) to (4 = very often). The total score of ASRS ranges between 0 and 72. Higher scores reflect higher ADHD psychopathology (Kessler et al., 2005; Brevik et al., 2020). Its reliability in the current sample is very good (coefficient alpha = 0.89).

The Internet Gaming Disorder Scale—Short-Form 9 (IGDS-SF9) is a brief measure of the severity of IGD symptoms (Pontes and Griffiths, 2015). The scale comprises nine items that are rated on a 5-point Likert scale ranging from (1 = never) to (5 = very often). The total score of the IGDS-SF9 ranges between 9 and 45. Higher scores reflect higher levels of problematic internet gaming (Pontes and Griffiths, 2015). Its reliability in the current sample is very good (coefficient alpha = 0.87).

The Individualism and Collectivism Scale/Culture Orientation Scale (ICS) comprises 16 items, in four dimensions of cultural orientation (Triandis and Gelfand, 1998), characterized as vertical, which emphasizes hierarchy and rankings or horizontal, which emphasizes equality: (1) vertical collectivism, the idea of complying with the group norms, group authority, general interest, and obligations instead of one’s own personal interest while supporting competition with out-groups, for example, “It is my responsibility to take care of my family, even when I have to sacrifice what I want”; (2) vertical individualism, the idea that everyone is in competition and winning or achievement-based rankings determine perceived value, for example, “Competition is the law of nature”; (3) horizontal collectivism, desire for being similar to others, sharing common goals with others, interdependence, and sociability, without the need for submission to authority, for example, “I feel good when I cooperate with others”; and (4) horizontal individualism, the idea of unique personality, self-reliance, and being distinct from groups, for example, “I’d rather depend on myself than others; my personal identity, independent of others, is very important to me” (Triandis and Gelfand, 1998; DeLuca et al., 2020; Germani et al., 2020). Each dimension comprises four items rated on a 9-point Likert scale (1 = never or definitely no) to (9 = always or definitely yes). The scores of items on each dimension are summed to provide an individual score of each dimension; the total score of each dimension ranges between 4 and 36. A higher score reflects greater alignment with the cultural context of the dimension. The reliability of the ICS in this study is acceptable (coefficient alpha = 0.67; Andreetta et al., 2020).

The IGDS-SF9, ASRS, and ICS were collected only from the English-speaking sample, and they were used to examine criterion validity of the DASS-21 and its tested short versions.

### Statistical Analysis

Because participants in both samples were healthy, they were merged together in one large international sample. We examined the distribution of the DASS-21 and its short versions using Shapiro–Wilk test. Quantitative variables with non-normal distribution were described using median and interquartile range (IQR: 25–75%) while qualitative variables were described using frequency and percentage. The factor structures of the DASS-12 and DASS-8 (Ali et al., 2021a) were examined in the whole international sample using CFA. Four models were tested: one-factor, three-factor, second-order factor, and bifactor structures. Because the distribution of the tested short versions of the DASS-21 was not normal (Supplementary Materials), the fit of the models testing the structures of these scales was examined *via* structural equation modeling involving maximum likelihood method of estimation with bootstrap that generates 2000 random samples. This is because Amos does not employ weighted least squares method of estimation. Meanwhile, analyzing non-normal data derived from large samples by maximum likelihood yields similar results to those attained by weighted least squares method of estimation—in terms of overall fit and the discrepancy between estimated parameter values and the true parameter values used to generate the data (Olsson et al., 2000).

Although a non-significant chi-square (*χ*^2^) index indicates perfect model-data fit, *χ*^2^ is largely dependent on sample size. Thus, well-fitting models with minor misspecifications may be disqualified based on *χ*^2^ (Ali et al., 2021c). Alternatively, absolute fit indices can more reliably reflect model fit because they are sample size independent. Hence, model fit was considered good or acceptable based on Comparative Fit Index (CFI) and Tucker–Lewis Index (TLI) equal to or above 0.95 and 0.90, respectively, along with root mean square error of approximation (RMSEA) and standardized root mean square residual (SRMR) less than 0.06 and 0.08, respectively (Ali et al., 2021d).

Using multigroup CFA, invariance of the three-factor structure of the DASS-8/DASS-12 across gender groups in the whole international sample, employment status (employed vs. non-employed) in the English-speaking sample, and countries (Australia, US, and Ghana) was evaluated at the configural, metric, scalar, and strict levels as described elsewhere (Ali et al., 2021c). This is because the number of participants from the United Kingdome, New Zealand, and other locations was very small, disqualifying their inclusion in invariance analysis. Because all Ghanian participants were students, and the literature documents higher levels of distress among students than the general population (Ali et al., 2021b), the structure of the DASS-8/DASS-12 was compared across English-speaking and Ghanian students as well as across English-speaking students and non-students. In nested model comparisons, invariance was depicted by significant *χ*^2^, along with ΔCFI and ΔRMSEA above 0.02 and 0.015, respectively (Ali et al., 2021b). For both CFA and multigroup CFA, model fit was improved by correlating error residuals suggested by modification indices reported for each model. Because of scalar variance of the DASS-12/DASS-8 across countries, Mann Whitney U-test was used to investigate the differences in the scores of both scales between English-speaking and Ghanian participants.

Discriminant validity of the DASS-21 and its shortened version was examined by the heterotrait–monotrait (HTMT) ratio of correlations (Henseler et al., 2015). For the DASS-21 and its shortened versions, McDonald’s Omega coefficient was estimated in CFA models, which comprised equally weighted phantom variable composite variables in addition to the scaling/identification specifications. Employing 2000 parametric bootstrapped replications, Amos was prompted to estimate the implied correlations between phantom variables and the corresponding variables covered by the scales (depression, anxiety, and stress) along with 95% CI, which were all then squared (Gignac, 2014). Additionally, coefficient alpha, alpha-if-item deleted, and item–total correlations were used to evaluate the reliability of the DASS-21, DASS-12, DASS-8, and their subscales in the whole sample. Criterion validity of the DASS-21, DASS-12, DASS-8, and their subscales was evaluated by Spearman’s rho correlations with the IGDS-SF9, ASRS, and its subscales, and subscales of the ICS in the English-speaking group only. SPSS and Amos were used for statistical analysis and significance was considered at a probability level less than 0.05 in two-tailed tests.

## Results

### Results of Confirmatory Factor Analysis

**Figure 1:**
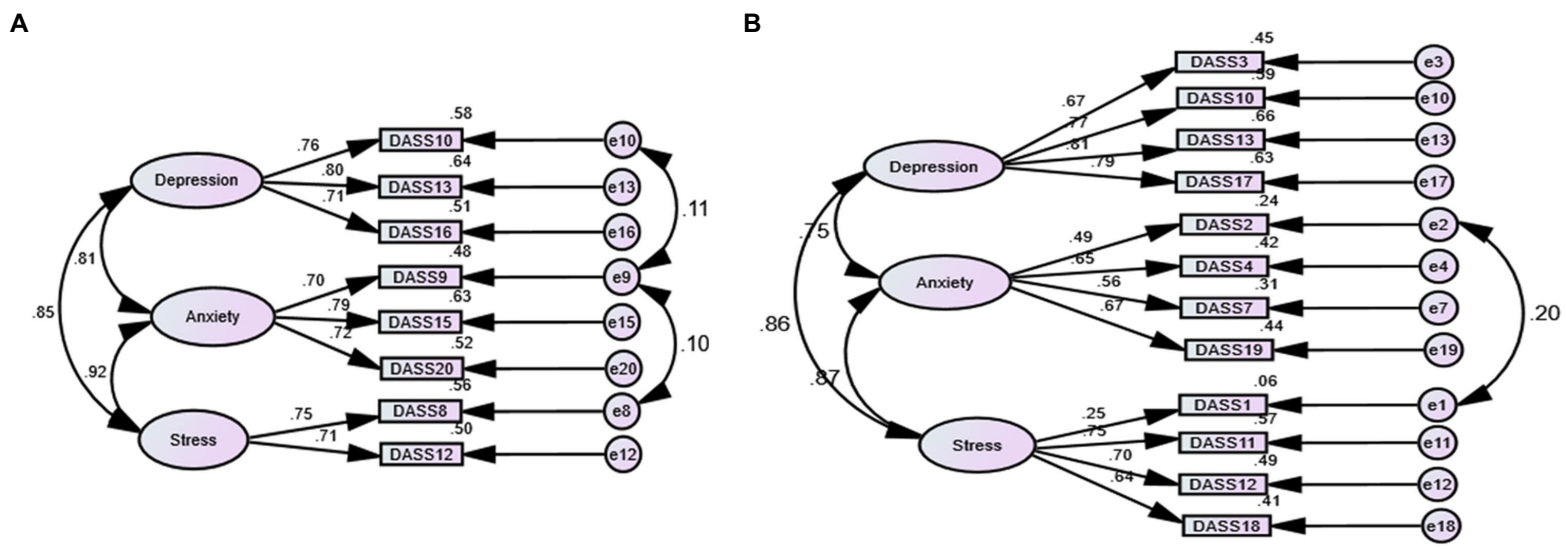
Three-factor structure of shortened versions of the Depression Anxiety stress scale (DASS) 21 in healthy participants, in order: the DASS-8 **(A)** and the DASS-12 **(B)**.

As shown in Table 1, the fit of the one-factor structures of the DASS-8/DASS-12 was acceptable, and it improved when some error terms were correlated (Supplementary Materials). The crude models of the three-factor structures of the DASS-8 and the DASS-12 expressed good fit on all indices. Correlating the error terms of few items (Figure 1) in both models slightly improved the fit. Figure 1 also shows that all items of the DASS-8 considerably contributed to the corresponding latent factors as noted by high loadings of all items (0.70 to 0.80). On the other hand, the loading of item 1 on the DASS-12 was below 0.3 while the loadings of most items were considerably lower than the loadings of items of the DASS-8 (0.49 to 0.81). The fit of the second-order factor was similar to the three-factor structures of the DASS-8 and the DASS-12 (Supplementary Materials). Meanwhile, the model representing the bifactor structure of the DASS-8 failed to converge until 85 outlier responses were eliminated. Model fit was similar to the three-factor structure with all items loading significantly on the corresponding domain-specific factors, but none of the items loaded on the general factor. Although all items of the DASS-12 loaded significantly (all values of *p* < 0.01) on the general factor, all items of the stress subscale failed to load on their corresponding stress factor (Supplementary Materials).

**Table 1:** Goodness-of-fit of the confirmatory factor analysis models representing the Depression Anxiety Stress Scale-8 (DASS-8) and the DASS-12 in the whole sample.

**Models**	**Samples**	***χ*** ^**2**^	***p***	***df***	**CFI**	**TLI**	**RMSEA**	**RMSEA 90% CI**	**SRMR**
Model 1	Crude	496.84	0.001	54	0.93	0.91	0.08	0.07 to 0.08	0.04
1F Korean DASS-12	Correlated error	322.41	0.001	51	0.96	0.94	0.06	0.06 to 0.07	0.03
Model 2	Crude	192.66	0.001	51	0.98	0.97	0.04	0.04 to 0.05	0.03
3F Korean DASS-12	Correlated error	140.35	0.001	50	0.99	0.99	0.04	0.03 to 0.04	0.02
Model 3 bifactor DASS-12	Crude	91.31	0.001	49	0.99	0.98	0.03	0.02 to 0.04	–
Model 4	Crude	270.70	0.001	20	0.95	0.93	0.09	0.08 to 0.10	0.04
1F DASS-8	Correlated error	121.94	0.001	17	0.98	0.97	0.07	0.06 to 0.08	0.03
Model 5	Crude	63.22	0.001	17	0.99	0.99	0.04	0.03 to 0.06	0.02
3F DASS-8	Correlated error	44.96	0.001	15	0.99	0.99	0.04	0.03 to 0.05	0.02
Model 6 bifactor DASS-8	Crude	54.71	0.001	16	0.99	0.98	0.04	0.03 to 0.06	–

*DASS, Depression anxiety stress scale; χ^2^, chi-square; df, degrees of freedom; CFI, Comparative fit index; TLI, Tucker–lewis index; RMSEA, Root mean square error of approximation; CI, Confidence interval; SRMR, Standardized root mean residual; –: SRMR was not estimated indicating model misfit*.

### Results of Invariance Analysis

**Figure 2:**
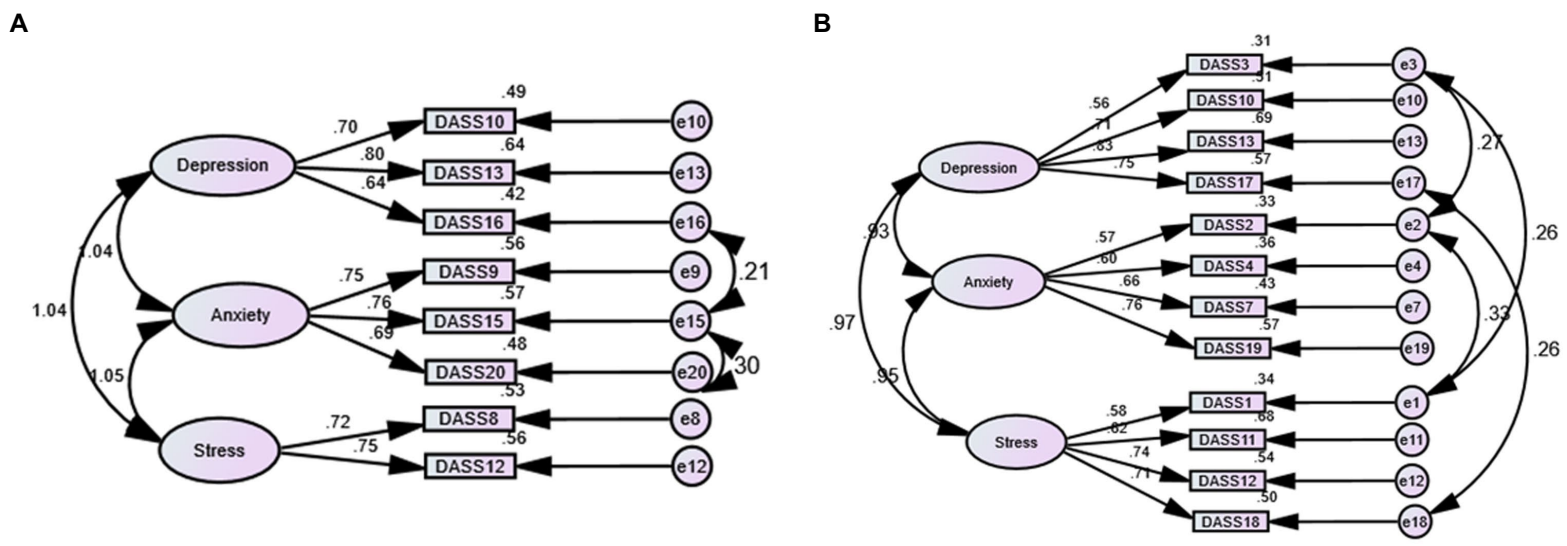
Three-factor structure of the Depression Anxiety stress scale (DASS) 8 **(A)** and the DASS-12 **(B)** expressing the best fit in participants from Ghana.

As shown in Supplementary Table 1, the crude models of the three-factor structures of the DASS-8 and DASS-12 were invariant across gender groups at the configural, metric, and scalar levels in the whole international sample—high values of ΔCFI and ΔTLI highlight a tendency toward variance at the strict level in both models, but ΔRMSEA was within the acceptable bound. Examination of each model among participants from different countries (Table 2) revealed misspecification of the factor loadings in participants from Ghana as noted by inflated RMSEA in both models, along with a considerable reduction in other model fit indices in the DASS-12. In the Ghanaian sample, correlating several error terms (Figure 2) slightly improved the fit of the DASS-12 (*χ*^2^(47) = 157.21, *p* = 0.001, CFI = 0.95, TLI = 0.93, RMSEA = 0.08, SRMR = 0.04) while correlating the error term of item 15 with those of item 16 and 20 considerably improved the model fit of the DASS-8 (*χ*^2^(15) = 41.18, *p* = 0.275, CFI = 0.98, TLI = 0.97, RMSEA = 0.07, SRMR = 0.03).

**Table 2:** Invariance of factor structures of the Depression Anxiety Stress Scale 8 (DASS-8) and DASS-12 across countries.

**Model**	**Invariance levels**	*χ* ^2^	**df**	***p***	**Δ** *χ* ^2^	**Δdf**	***p*(Δ*χ*** ^**2**^ **)**	**CFI**	**ΔCFI**	**TLI**	**ΔTLI**	**RMSEA**	**ΔRMSEA**	**SRMR**
DASS-8	Australia	28.05	15	0.021				0.99		0.99		0.03		0.02
	US	28.89	15	0.017				0.99		0.98		0.07		0.03
	Ghana	64.84	15	0.000				0.97		0.94		0.09		0.03
	Configural	121.83	45	0.000				0.98		0.97		0.04		0.02
	Metric	146.36	55	0.000	24.53	10	0.006	0.98	0.00	0.97	0.00	0.04	0.00	0.02
	Strong	266.94	67	0.000	120.06	12	0.001	0.96	**0.03**	0.95	**0.02**	0.05	−0.01	0.04
	Strict	453.68	87	0.000	186.74	20	0.001	0.92	**0.06**	0.93	**0.05**	0.06	**0.02**	0.06
Korean DASS-12	Australia	76.61	50	0.009				0.99		0.98		0.03		0.03
	US	69.35	50	0.036				0.98		0.98		0.04		0.04
	Ghana	215.54	50	0.000				0.93		0.91		0.09		0.05
	Configural	361.66	150	0.000				0.96		0.95		0.03		0.03
	Metric	456.63	168	0.000	94.98	18	0.006	0.95	0.01	0.94	0.01	0.04	−0.01	0.04
	Strong	609.40	180	0.000	152.77	12	0.001	0.93	**0.04**	0.92	**0.03**	0.04	−0.01	0.06
	Strict	1051.87	206	0.000	442.47	26	0.001	0.85	**0.11**	0.86	**0.09**	0.056	**−0.02**	0.07

*DASS, Depression anxiety stress scale; χ^2^, chi-square; df, degrees of freedom; CFI, Comparative fit index; TLI, Tucker–Lewis index; RMSEA, Root mean square error of approximation; CI, Confidence interval; SRMR, Standardized root mean residual; values in boldface denote tendency to variance*.

Because students tend to express more stress than the general population (Ali et al., 2021b), we compared the DASS-8/DASS-12 across Ghanaian and English-speaking students. According to Table 3, the DASS-8/DASS-12 tended to express variance at the scalar level and were variant at the strict level across Ghanaian and English-speaking student groups. Examination of the difference in mental symptomatology levels in both student groups (Table 4) uncovered statistical differences in the total scores of the DASS-21, DASS-12, DASS-8, and their subscales between Ghanaian and English-speaking students. The latter scored higher on all the measures compared with Ghanian students. To determine if the mean scores attained by DASS-8/DASS-12 are affected by the state of being a student, we examined invariance of both measures across student and non-student participants in the English-speaking sample. As illustrated in Supplementary Table 2, the DASS-8/DASS-12 were invariant at the configural, metric, scalar, and strict levels.

**Table 3:** Invariance of factor structures of the Depression Anxiety Stress Scale 8 (DASS-8) and DASS-12 across English-speaking and Ghanaian student groups.

**Model**	**Invariance levels**	*χ* ^2^	**df**	***P***	**Δ** *χ* ^2^	**Δdf**	***p*(Δ** *χ* ^2^ **)**	**CFI**	**ΔCFI**	**TLI**	**ΔTLI**	**RMSEA**	**ΔRMSEA**	**SRMR**
DASS-8	English-speaking	30.83	17	0.021				0.99		0.98		0.05		0.03
	Ghanaian	84.93	17	0.001				0.96		0.93		0.1		0.04
	Configural	115.75	34	0.001				0.97		0.95		0.06		0.04
	Metric	122.09	39	0.001	6.34	5	0.275	0.97	0	0.96	-0.01	0.05	0.01	0.04
	Strong	209.06	45	0.001	86.97	6	0.001	0.94	**0.03**	0.93	**0.03**	0.07	0.02	0.05
	Strict	310.82	53	0.001	101.77	8	0.001	0.91	**0.03**	0.91	**0.02**	0.08	−**0.01**	0.06
Korean DASS-12	English-speaking	91.55	51	0.036				0.97		0.96		0.05		0.04
	Ghanaian	250.1	51	0.001				0.91		0.89		0.1		0.05
	Configural	342.52	102	0.001				0.94		0.92		0.06		0.05
	Metric	390.18	111	0.001	94.98	18	0.006	0.93	0.01	0.91	0.01	0.06	−0.00	0.06
	Strong	472.2	117	0.001	152.77	12	0.001	0.9	**0.02**	0.89	**0.02**	0.06	−0.00	0.07
	Strict	800.36	129	0.001	442.47	26	0.001	0.82	**0.09**	0.81	**0.08**	0.08	**0.02**	0.11

*DASS, Depression anxiety stress scale; χ^2^, chi-square; df, degrees of freedom; CFI, Comparative fit index; TLI, Tucker–Lewis index; RMSEA, Root mean square error of approximation; CI, Confidence interval; SRMR, Standardized root mean residual; values in boldface denote tendency to variance*.

**Table 4:** Descriptive statistics of and between-group differences in the scores of the Depression Anxiety Stress Scale (DASS) 21, DASS-12, DASS-8, and their subscales among English-speaking and Ghanian students.

**DASS subscales**	**Samples**	**DASS-21**					**DASS-12**					**DASS-8**				
		***MD***	***Q1***–***Q3***	***U***	***W***	***z***	***MD***	***Q1***–***Q3***	***U***	***W***	***z***	***MD***	***Q1***–***Q3***	***U***	***W***	***z***
**Depression**	English	8	3–12	37719.5	114747.5	−10.42	4	2–7	39417.5	116445.5	−9.92	3	1–5	41124.5	118152.5	−9.36
	Ghanaian	3	0–6				1	0–3				1	0–3			
**Anxiety**	English	6	2–9	42323.5	119351.5	−8.82	3	1–5	42095.5	119123.5	−9.05	3	1–5	48183.0	125211.0	−6.85
	Ghanaian	2	0–6				1	0–3				1	0–3			
**Stress**	English	8	4–11	35092.5	112120.5	−11.32	4	2–6	35651.5	112679.5	−11.18	2	1–3	39593.5	116621.5	−10.02
	Ghanaian	3	0–7				2	0–4				0	0–2			
**Whole scale**	English	22	11–31	35225.5	112253.5	−11.24	12	6–17	34191.0	111219.0	−11.62	9	4–13	39073.0	116101.0	−9.94
	Ghanaian	8	2–18				4	1–10				3	0–7			

*DASS: Depression anxiety stress scale; MD, median; Q1, first quartile, Q3, third quartile; U, Mann–Whitney U-test, W, Wilcoxon test, all values of p < 0.001*.

### Discriminant Validity, Internal Consistency, and Criterion Validity

As shown in the Supplementary Excel file, the three-factor structure of the DASS-21, which comprised some correlated error terms, had good fit. However, the HTMT ratios of correlations of the depression, anxiety, and stress constructs were all above 0.85, indicating that the constructs measured by the DASS-21 are not really distinct from one another. The DASS-12 could differentiate depression from anxiety (HTMT ratios = 0.75), but it could not distinguish depression or anxiety from stress (HTMT ratios = 0.92 and 0.88). As for the DASS-8, HTMT ratios were all below 0.85 except for the stress–anxiety scales, indicating that these two subscales are not quite discrete.

Table 5 presents the internal consistency of the DASS-21 and its short versions, highlighting that the reliability of the DASS-8, as well as its anxiety and stress subscales, was comparable with or even better than that of the DASS-12 and its anxiety and stress subscales. The DASS-8 and its subscales also displayed high item–total correlations—comparable with the parent scale and higher than the DASS-12. The correlations of the DASS-8 and its subscales with the DASS-21 and its subscales were all high (*p* < 0.001). As we hypothesized, criterion validity testing in the English-speaking sample uncovered strong positive correlation of the DASS-21 and its short versions with the scores of IGDS-SF9, ASRS, and its inattention and impulsivity–hyperactivity subscales. Contrary to expectations, all the DASS measures (except for the anxiety subscale on the DASS-21 and the DASS-12 and the depression subscale on the DASS-8) had moderate positive correlation with the individualism subscales and negative correlation with the collectivism subscales of the ICS, discussed in detail below.

**Table 5:** Internal consistency and criterion validity of the Depression Anxiety Stress Scale (DASS) 21, DASS-12, DASS-8, and their subscales among the participants.

Criteria	**DASS-21**	**Depression**	**Anxiety**	**Stress**	**DASS-12**	**Depression**	**Anxiety**	**Stress**	**DASS-8**	**Depression**	**Anxiety**	**Stress**
Coefficient alpha	0.93	0.90	0.82	0.81	0.87	0.85	0.67	0.66	0.89	0.80	0.78	0.69
Range of corrected item-total correlations	0.24–0.74	0.60–0.75	0.38–0.67	0.23–0.67	0.47–0.71	0.61–0.72	0.37–0.52	0.21–0.57	0.62–0.70	0.63–0.67	0.59–0.69	0.53
Range of alpha if-item-deleted	0.93–0.94	0.87–0.89	0.77–0.82	0.77–0.84	0.86–0.87	0.79–0.83	0.57–0.67	0.50–0.74	0.867–0.875	0.718–0.756	0.679–0.736	–
McDonald’s Omega coefficient	0.94	0.90	0.82	0.82	0.87	0.85	0.68	0.68	0.87	0.80	0.78	0.69
McDonald’s Omega coefficient 95% CI	0.93–0.94	0.89–0.91	0.80–0.84	0.80–0.83	0.85–0.88	0.83–0.86	0.64–0.71	0.66–0.71	0.85–0.88	0.79–0.82	0.76–0.80	0.65–0.73
Correlation with the corresponding subscale on the DASS-21	–	–	–	–	–	0.96^***^	0.89^***^	0.93^***^	–	0.94^***^	0.90^***^	0.85^***^
Correlation with the DASS-21	–	0.92^***^	0.89^***^	0.91^***^	0.97^***^	0.89^***^	0.78^***^	0.83^***^	0.95^***^	0.87^***^	0.82^***^	0.80^***^
Correlation with IGDS-SF9	0.44^**^	0.41^**^	0.34^**^	0.41^**^	0.42^**^	0.39^**^	0.28^**^	0.35^**^	0.40^**^	0.37^**^	0.32^**^	0.34^**^
Correlation with ASRS	0.56^**^	0.46^**^	0.52^**^	0.54^**^	0.53^**^	0.41^**^	0.46^**^	0.48^**^	0.52^**^	0.40^**^	0.46^**^	0.48^**^
Correlation with inattention	0.55^**^	0.46^**^	0.49^**^	0.50^**^	0.52^**^	0.42^**^	0.43^**^	0.44^**^	0.49^**^	0.41^**^	0.43^**^	0.44^**^
Correlation with impulsivity-hyperactivity	0.51^**^	0.39^**^	0.47^**^	0.51^**^	0.48^**^	0.36^**^	0.41^**^	0.46^**^	0.47^**^	0.35^**^	0.41^**^	0.47^**^
Correlation with horizontal individualism	0.13^**^	0.09^*^	0.02	0.13^**^	0.07^*^	0.08^*^	−0.01	0.11^**^	0.09^*^	0.05	0.10^*^	0.09^*^
Correlation with vertical individualism	0.17^**^	0.11^**^	0.13^**^	0.23^**^	0.15	0.10^*^	0.11^**^	0.20^**^	0.19^**^	0.11^**^	0.11	0.17^**^
Correlation with horizontal collectivism	−0.17^**^	−0.22^**^	−0.12^**^	−0.16^**^	−0.18^**^	−0.18^**^	−0.12^**^	−0.13^**^	−0.18^**^	−0.22^**^	−0.10^*^	−0.13^**^
Correlation with vertical collectivism	−0.22^**^	−0.23^**^	−0.18^**^	−0.17^**^	−0.21^**^	−0.21^**^	−0.13^**^	−0.16^**^	−0.20^**^	−0.20^**^	−0.14^**^	−0.15^**^

IGDS-SF9, Internet gaming disorder scale—short-form 9; ASRS, Adult attention-deficit/hyperactivity disorder Self-Report Scale; ^*^, ^**^, and ^***^: correlation is significant at 0.05, 0.01, and 0.001 levels. Spearman’s rho test for criterion validity was conducted only in the English-speaking group.

## Discussion

This study enriches the current knowledge by examining the psychometric properties of the DASS-8, the briefest version of the DASS-21, relative to the DASS-12 in an international sample from three countries with different cultural orientations. Both the DASS-8 and DASS-12 are best expressed by a three-factor structure. The DASS-8 expressed good fit, with all items considerably loading on the corresponding factors. On the contrary, the loadings of most items of the DASS-12 were remarkably lower, particularly item 1. As hypothesized, the DASS-8 demonstrated invariance across gender and employment groups—it was more likely to be invariant across countries relative to the DASS-12. It also expressed other robust psychometric characteristics.

The correlation of the DASS-12, the DASS-8, and their subscales with the DASS-21 and its subscales were high, denoting adequate item coverage and predictive validity. The internal consistency and item–total correlations of the DASS-8 and its subscales were relatively higher than the DASS-12. Of interest, the anxiety and stress subscales of the DASS-12 expressed poorer reliability though they contained 25 and 50% more items than the corresponding subscales of the DASS-8 (Table 5). Unlike the DASS-8, alpha-if-item deleted indicated that the reliability of the stress subscale of the DASS-12 would increase if some items were removed (Table 5). This result is congruent with the low loadings of several items on this subscale—item 1 had a loading below 0.3 denoting that it does not contribute to the stress construct measured by the DASS-12 or the DASS-21 (Figure 1B; Supplementary Excel). Discriminant validity testing revealed overlapping of the constructs measured by the DASS-21 (HTMT ratios >0.85). This finding is consistent with investigations reporting failure of the DASS-21 to distinguish depression from anxiety or stress, denoting the suitability of the scale to be used as a measure of psychological distress (Osman et al., 2012; Zanon et al., 2020). On the contrary, the shorter versions of the scale demonstrated discrete identities of the depression and anxiety constructs. Nonetheless, the stress subscale was not distinct from the depression and the anxiety constructs depicted by the DASS-12. Among all the constructs covered by the DASS-8, stress was overlapping only with the construct of anxiety. Therefore, the DASS-8, unlike the parent DASS-21 and the DASS-12, can be best used to represent mental symptomatology of depression, anxiety, and stress as distinct/independent constructs. As noted by these results, high reliability, predictive validity, discriminant validity, and convergent validity of the DASS-8 reflect high specificity and sensitivity of its items, which is in line with a previous testing of the DASS-8 among healthy individuals and psychiatric patients from Arab countries (Ali et al., 2021a).

The DASS-8 and its subscales correlated with variables used for criterion validity testing at the same level of significance expressed by the parent scale and the DASS-12 or even higher (e.g., horizontal individualism, Table 5). In more details, the DASS-8 positively correlated with measures of IGD and ADHD as we hypothesized. This finding suggests usefulness of the DASS-8 as a criterion variable that can significantly predict addictive pathologies and mental disorders. Consistent with our results, depression and anxiety symptoms significantly predicted ADHD in community-dwelling youth, and emotional regulation significantly mediated this relation (Seymour et al., 2014). Likewise, previous investigations in the current English-speaking sample show that the stress subscale of the DASS-21 significantly predicted IGD, especially among men who had high scores on vertical individualism (Andreetta et al., 2020). Our analysis also shows that the DASS scales positively correlated with vertical and horizontal individualism and negatively correlated with collectivism scales, which is inconsistent with our priori hypothesis. This finding is further illustrated below within the context of invariance of the DASS-8/DASS-12 across countries.

The DASS-8 and the DASS-12 were invariant across groups of gender in the international sample and across student and non-student English-speaking participants. Nonetheless, English-speaking participants (both students and non-students) in the current study scored higher on all the mental symptomatology scales compared with Ghanian students as noted by invariance of the DASS-8/DASS-12 at the scalar level and significant Mann–Whitney U-test for between-group differences (Table 3, 4). This result is congruent with former investigations reporting variance of the DASS-21 at the scalar level across countries (Scholten et al., 2017; Bibi et al., 2020; Zanon et al., 2020). However, in previous studies, English-speaking respondents reported lower levels of anxiety and stress compared with respondents from Russia and Poland (Scholten et al., 2017). Likewise, European respondents from Germany reported lower levels of anxiety and depression compared with respondents from Pakistan, a relatively poor Asian country (Bibi et al., 2020).

It is worth mentioning that data from our English-speaking participants were collected before the COVID-19 pandemic while data were obtained from the Ghanian sample during the COVID-19 outbreak. This period has witnessed an inflation in the rates of negative emotions all over the world (Ali et al., 2021e). Although there is no clear explanation for invariance of the DASS-8/DASS-12 across English-speaking and Ghanian respondents in the present study, criterion validity tests indicate that this finding can be attributed to cultural differences—higher vertical individualism in the English-speaking samples. On the other hand, the literature reports a collectivistic nature of most countries in Africa, including Ghana (Letseka and Maile, 2008; Sweetland et al., 2014; Anlesinya et al., 2019), albeit cultural orientation was not examined in the current Ghanian sample.

Systematic investigations of emotional negativity in sub-Saharan Africa report differences in the salience, manifestation, and expression of symptoms; despite evidence of the underlying universality in the experience of depression and anxiety in that region (Sweetland et al., 2014). Given the contribution of cultural contexts to the experience and expression of emotions (Office of the Surgeon General (US) et al., 2001; Sweetland et al., 2014; Megreya et al., 2018), low levels of distress among Ghanian participants despite the pandemic can be attributed to the widespread of destiny orientation in Ghana. Destiny orientation describes a tendency to perceive events as determined by external factors (e.g., luck, gods, and spirits); thus, events are accepted to be out of control or cannot be changed by human action (Nordfjærn et al., 2012). Considerably, more than two-thirds the Ghanian participants were females. University students in most African countries, particularly females, depend on their parents or guardians for financial support to pay their tuition fees and support their essential living expenses (Letseka and Maile, 2008). Although the Ghanian society is predominantly masculine and collectivistic, women’ s perceived support system is reported to buffer the negative consequences of cultural power distance in Ghana (Anlesinya et al., 2019), which may justify low levels of distress among our Ghanian students. On the contrary, many students in Western countries do part-time jobs to support their education and living, which may be an extra burden that leads to increased distress symptomatology (Ali et al., 2021b). In support of this view, 47.0% (*N* = 162) of our English-speaking students performed some sort of employment. Moreover, tests of criterion validity show that all the DASS measures and their subscales in the English-speaking sample positively correlated with vertical individualism (Table 5), which is primarily focused on competitiveness and achievement as bases for achieving social hierarchies (Andreetta et al., 2020).

In the general population, competitiveness is associated with increased risk for mortality from cardiovascular diseases (Lohse et al., 2017), entailing dysregulation of the hypothalamic pituitary adrenal axis (HPA) in highly competitive individuals. HPA plays a role in the regulation of stress and emotions. According to the cultural transactional theory of stress and coping, stress evolves from transactions taking place between a person and the complex environment. Through primary appraisal, individuals appraise the extent to which stressors represent threats while secondary appraisal involves evaluation of individuals’ internal and external resources. Individuals with adequate resources to handle the stressors apply problem-focused coping (primary control). On the other side, individuals use emotion-focused coping (secondary control) when they encounter overwhelming challenges that are beyond their capacity to manage (Shekriladze et al., 2021). During the COVID-19 lockdown, individualism and collectivism moderated the association between anxiety and passive–submissive coping styles (Shekriladze et al., 2021). Having self as a core unit of the society in individualistic orientations entails high priority of individual achievement and autonomy (Shekriladze et al., 2021). Individualistic people express a higher tendency to social compliance to the perceived social hierarchy (Pontes et al., 2017). However, when socializing to divergent cultural orientations is viewed as a challenge, it may increase individualistic individuals’ tendency to self-blame and guilt as well as their experience of depression (Pontes et al., 2017; Mumang et al., 2021). In fact, perceived contextual competitiveness for achievement and social status is associated with poor social cohesion and chronic stress (Melita et al., 2021). In line, a previous investigation involving the present English-speaking sample reports that depression symptoms among men and women high on vertical individualism are associated with higher levels of disordered gaming behaviors (O’Farrell et al., 2020). Overall, the DASS-8 may effectively identify individuals high on emotion coping as a function of their cultural orientation. Thus, it may signal individuals in need for stress management interventions in order to prevent morbidity and dysfunctional behaviors.

This study has the merit of being the first to compare the psychometric properties of the DASS-8 to those of the DASS-12 across different cultural contexts. The findings confirm usability of the DASS-8 as a measure of depression, anxiety, and stress symptoms in English-speaking participants from different countries (the US and Australia) as well as in participants from sub-Saharan Africa, regardless variations in their levels of distress. The study has also several limitations. The cross-sectional design precludes evaluating test–retest reliability. The use of online survey for data collection as well as lack of information on the specific sampling strategy used, response rate, and further details on the inclusion/exclusion criteria entail a possible risk for selection bias. Meanwhile, power analysis was not used to estimate sample sizes. Some of the sociodemographic characteristics of the Ghanian participants were lacking, which prevented comparing the DASS-8 across various basic characteristics, for example, working status or living situation (e.g., alone or with family). Although Mann–Whitney test revealed higher levels of distress among participants from Australia and the US than Ghanian participants, it is necessary for future studies to test whether the DASS-8 can differentiate healthy individuals from those with depression/anxiety psychopathology in order to confirm the discriminant validity of the scale. Although weighted least square method of estimation is more robust than maximum likelihood in case of non-normal data (Olsson et al., 2000), the later was used in the present study because the former is not estimated in Amos. However, the sample size was relatively big, and with a bootstrap involving 2000 random replications, the difference between both methods should be minimal (Olsson et al., 2000), albeit the possibility that the results are bias-free may not be ensured. Thus, further investigations of the characteristics of these short versions of the DASS-21 are needed.

## Conclusion

Our results confirm previously reported soundness of the psychometric properties of the DASS-8 such as good fit and invariance across gender groups and across students and non-students from English-speaking countries. The tendency of the DASS-8/DASS-12 to vary across Ghanian and English-speaking participants reflects lower levels of distress in the Ghanian sample, which may be attributed to cultural differences that affect the experience and expression of mental symptomatology. The internal consistency of the DASS-8 and most of its subscale was higher than the DASS-12, denoting that despite the extensive reduction of its items, the DASS-8 retained the most specific and sensitive items to the relevant latent construct. Thus, the DASS-8 may be used in clinical practice and research to identify individuals at risk for psychopathology or evaluate response to treatment. Further investigation, especially in disordered groups from more diverse cultures, is warranted.

## Data Availability Statement

The datasets presented in this study can be found in online repositories. The names of the repository/repositories and accession number(s) can be found in the article.

## Ethics Statement

Ethical review and approval was not required for the study on human participants in accordance with the local legislation and institutional requirements—previously available public data were used in the analysis. The participants provided their written informed consent to complete the data collection questionnaires.

## Author Contributions

AMA and HK conceptualized the topic. YK and HH acquired fund. AMA and HH conducted the statistical analysis and wrote the first draft of the manuscript. HK and YK reviewed and edited the manuscript. All authors contributed to the article and approved the submitted version.

## Funding

This study was partially supported by the National Center Cohort Collaborative for Advancing Population Health funded by the Japan Health Research Promotion Bureau (JH) Research Fund (Project Number 2019-(1)-1) and by JSPS KAKENHI (Grant Number: 20K07937).

## Conflict of Interest

The authors declare that the research was conducted in the absence of any commercial or financial relationships that could be construed as a potential conflict of interest.

## Publisher’s Note

All claims expressed in this article are solely those of the authors and do not necessarily represent those of their affiliated organizations, or those of the publisher, the editors and the reviewers. Any product that may be evaluated in this article, or claim that may be made by its manufacturer, is not guaranteed or endorsed by the publisher.

## Supplementary Material

The Supplementary Material for this article can be found online at: https://www.frontiersin.org/articles/10.3389/fpsyg.2022.799769/full#supplementary-material

## References

[ref1] Ali A. M. Alkhamees A. A. Hori H. Kim Y. Kunugi H. (2021a). The depression anxiety stress scale 21: development and validation of the depression anxiety stress scale 8-item in psychiatric patients and the general public for easier mental health measurement in a post-COVID-19 world. Int. J. Environ. Res. Public Health 18:10142. doi: 10.3390/ijerph181910142, PMID: 34639443 PMC8507889

[ref2] Ali A. M. Green J. (2019). Factor structure of the depression anxiety stress Scale-21 (DASS-21): Unidimensionality of the Arabic version among Egyptian drug users. Subst. Abuse Treat. Prev. Policy 14:40. doi: 10.1186/s13011-019-0226-1, PMID: 31533766 PMC6751677

[ref3] Ali A. M. Hendawy A. O. Ahmad O. Sabbah H. A. Smail L. Kunugi H. (2021b). The Arabic version of the Cohen perceived stress scale: factorial validity and measurement invariance. Brain Sci. 11:419. doi: 10.3390/brainsci11040419, PMID: 33810322 PMC8066085

[ref4] Ali A. M. Hendawy A. O. Almarwani A. M. Alzahrani N. Ibrahim N. Alkhamees A. A.. (2021c). The six-item version of the internet addiction test: its development, psychometric properties, and measurement invariance among women with eating disorders and healthy school and university students. Int. J. Environ. Res. Public Health 18:12341. doi: 10.3390/ijerph182312341, PMID: 34886068 PMC8657305

[ref5] Ali A. M. Hori H. Kim Y. Kunugi H. (2021d). Predictors of nutritional status, depression, internet addiction, Facebook addiction, and tobacco smoking among women with eating disorders in Spain. Front. Psychiatry. 12:2001. doi: 10.3389/fpsyt.2021.735109, PMID: 34899416 PMC8663168

[ref6] Ali A. M. Kunugi H. Abdelmageed H. A. Mandour A. S. Ahmed M. E. Ahmad S.. (2021e). Vitamin K in COVID-19—potential anti-COVID-19 effects of vitamin K antagonists (VKA) and fermented milk fortified with bee honey as a natural source of vitamin K and probiotics. Fermentation 7:202. doi: 10.3390/fermentation7040202

[ref7] Andreetta J. Teh M. J. Burleigh T. L. Gomez R. Stavropoulos V. (2020). Associations between comorbid stress and internet gaming disorder symptoms: are there cultural and gender variations? Asia Pac. Psychiatry 12:e12387. doi: 10.1111/appy.12387, PMID: 32286004

[ref8] Anlesinya A. Adepoju O. A. Richter U. H. (2019). Cultural orientation, perceived support and participation of female students in formal entrepreneurship in the sub-Saharan economy of Ghana. Int. J. Gend. Entrep. 11, 299–322. doi: 10.1108/IJGE-01-2019-0018

[ref9] Bibi A. Lin M. Zhang X. C. Margraf J. (2020). Psychometric properties and measurement invariance of depression, anxiety and stress scales (DASS-21) across cultures. Int. J. Psychol. 55, 916–925. doi: 10.1002/ijop.12671, PMID: 32253755

[ref10] Brevik E. J. Lundervold A. J. Haavik J. Posserud M. B. (2020). Validity and accuracy of the adult attention-deficit/hyperactivity disorder (ADHD) self-report scale (ASRS) and the Wender Utah rating scale (WURS) symptom checklists in discriminating between adults with and without ADHD. Brain Behav. 10:e01605. doi: 10.1002/brb3.1605, PMID: 32285644 PMC7303368

[ref11] Burleigh TL. (2019). IGD_Culture. Data Archiving and Networked Services (DANS) V1. doi:10.17026/dans-xgs-hpdn

[ref12] Caetano A. C. Oliveira D. Gomes Z. Mesquita E. Rolanda C. (2017). Psychometry and Pescatori projective test in coloproctological patients. Ann. Gastroenterol. 30, 433–437. doi: 10.20524/aog.2017.0145, PMID: 28655980 PMC5479996

[ref13] Cooke J. E. Eirich R. Racine N. Madigan S. (2020). Prevalence of posttraumatic and general psychological stress during COVID-19: a rapid review and meta-analysis. Psychiatry Res. 292:113347. doi: 10.1016/j.psychres.2020.113347, PMID: 32763477 PMC7392847

[ref14] Corcoran M. McNulty M. (2018). Examining the role of attachment in the relationship between childhood adversity, psychological distress and subjective well-being. Child Abuse Negl. 76, 297–309. doi: 10.1016/j.chiabu.2017.11.012, PMID: 29175733

[ref15] DeLuca J. S. Hwang J. Stepinski L. Yanos P. T. (2020). Understanding explanatory mechanisms for racial and ethnic differences in mental health stigma: the role of vertical individualism and right-wing authoritarianism. J. Ment. Health 31, 39–49. doi: 10.1080/09638237.2020.1836556, PMID: 33112173

[ref16] Drapeau A Marchand A Beaulieu-Prévost D. (2012). “Epidemiology of psychological distress,” in: Mental Illnesses-Understanding, Prediction and Control. Croatia, ed. Labate L., IntechOpen 105–134.

[ref17] Dyrbye L. N. Thomas M. R. Shanafelt T. D. (2006). Systematic review of depression, anxiety, and other indicators of psychological distress among U.S. and Canadian medical students. Acad. Med. 81, 354–373. doi: 10.1097/00001888-200604000-00009, PMID: 16565188

[ref18] Germani A. Delvecchio E. Li J.-B. Mazzeschi C. (2020). The horizontal and vertical individualism and collectivism scale: early evidence on validation in an Italian sample. J. Child Fam. Stud. 29, 904–911. doi: 10.1007/s10826-019-01571-w

[ref19] Gignac G. E. (2014). Fluid intelligence shares closer to 60% of its variance with working memory capacity and is a better indicator of general intelligence. Intelligence 47, 122–133. doi: 10.1016/j.intell.2014.09.004

[ref20] Gloster A. T. Rhoades H. M. Novy D. Klotsche J. Senior A. Kunik M.. (2008). Psychometric properties of the depression anxiety and stress Scale-21 in older primary care patients. J. Affect. Disord. 110, 248–259. doi: 10.1016/j.jad.2008.01.023, PMID: 18304648 PMC2709995

[ref21] Guerrini Usubini A. Cattivelli R. Varallo G. Castelnuovo G. Molinari E. Giusti E. M.. (2021). The relationship between psychological distress during the second wave lockdown of COVID-19 and emotional eating in Italian young adults: The mediating role of emotional Dysregulation. J. Person. Med.:569, 11. doi: 10.3390/jpm11060569 PMC8235082 34204480

[ref22] Henseler J. Ringle C. M. Sarstedt M. (2015). A new criterion for assessing discriminant validity in variance-based structural equation modeling. J. Acad. Mark. Sci. 43, 115–135. doi: 10.1007/s11747-014-0403-8

[ref23] Janiri D. Moser D. A. Doucet G. E. Luber M. J. Rasgon A. Lee W. H.. (2020). Shared Neural Phenotypes for Mood and Anxiety Disorders: a Meta-analysis of 226 Task-Related Functional Imaging Studies. JAMA Psychiatry 77, 172–179. doi: 10.1001/jamapsychiatry.2019.3351 31664439 PMC6822098

[ref24] Jun D. Johnston V. Kim J. M. O'Leary S. (2018). Cross-cultural adaptation and validation of the Depression, Anxiety and Stress Scale-21 (DASS-21) in the Korean working population. Work 59, 93–102. doi: 10.3233/wor-172661, PMID: 29439379

[ref25] Kessler R. C. Adler L. Ames M. Demler O. Faraone S. Hiripi E.. (2005). The World Health Organization adult ADHD self-report scale (ASRS): a short screening scale for use in the general population. Psychol. Med. 35, 245–256. doi: 10.1017/s0033291704002892, PMID: 15841682

[ref26] Le M. T. H. Tran T. D. Holton S. Nguyen H. T. Wolfe R. Fisher J. (2017). Reliability, convergent validity and factor structure of the DASS-21 in a sample of Vietnamese adolescents. PLoS One 12:e0180557. doi: 10.1371/journal.pone.0180557, PMID: 28723909 PMC5516980

[ref27] Lee E. H. Moon S. H. Cho M. S. Park E. S. Kim S. Y. Han J. S.. (2019). The 21-item and 12-item versions of the depression anxiety stress scales: psychometric evaluation in a Korean population. Asian. Nurs. Res. 13, 30–37. doi: 10.1016/j.anr.2018.11.006, PMID: 30503903

[ref28] Letseka M. Maile S.. High university drop-out rates: a threat to South Africa’s future: The Human Sciences Research Council (2008).

[ref29] Lohse T. Rohrmann S. Richard A. Bopp M. Faeh D. (2017). Type A personality and mortality: competitiveness but not speed is associated with increased risk. Atherosclerosis 262, 19–24. doi: 10.1016/j.atherosclerosis.2017.04.016 28478195

[ref30] Lovibond P. F. Lovibond S. H. (1995). The structure of negative emotional states: comparison of the depression anxiety stress scales (DASS) with the Beck depression and anxiety inventories. Behav. Res. Ther. 33, 335–343. doi: 10.1016/0005-7967(94)00075-U, PMID: 7726811

[ref31] Lovibond PF Lovibond SH. (1995). Manual for the Depression Anxiety Stress Scales. 2nd Edn. Sydney: Psychology Foundation.

[ref32] Mahmoud S. R. J. Hall L. E. (2010). The psychometric properties of the 21-item depression anxiety stress scale (DASS-21) among a sample of young adults. Southern online. J. Nurs. Res. 10, 21–34.

[ref33] Masuyama A. Kubo T. Sugawara D. Chishima Y. (2021). Interest consistency can buffer the effect of COVID-19 fear on psychological distress. Int. J. Ment. Heal. Addict., 1–12. doi: 10.1007/s11469-021-00564-5, PMID: 34220383 PMC8238385

[ref34] Mazza C. Ricci E. Biondi S. Colasanti M. Ferracuti S. Napoli C.. (2020). A Nationwide survey of psychological distress among Italian people during the COVID-19 pandemic: immediate psychological responses and associated factors. Int. J. Environ. Res. Public Health 17:3165. doi: 10.3390/ijerph17093165 PMC7246819 32370116

[ref35] Megreya A. M. Latzman R. D. Al-Emadi A. A. Al-Attiyah A. A. (2018). An integrative model of emotion regulation and associations with positive and negative affectivity across four Arabic speaking countries and the USA. Motiv. Emot. 42, 566–575. doi: 10.1007/s11031-018-9682-6

[ref36] Melita D. Willis G. B. Rodríguez-Bailón R. (2021). Economic inequality increases status anxiety Through perceived contextual competitiveness. Front. Psychol. 12:637365. doi: 10.3389/fpsyg.2021.637365, PMID: 34108908 PMC8182636

[ref37] Mumang A. A. Syamsuddin S. Maria I. L. Yusuf I. (2021). Gender differences in depression in the general population of Indonesia: confounding effects. Depress. Res. Treat. 2021, 3162445–3162448. doi: 10.1155/2021/3162445, PMID: 34258060 PMC8253638

[ref38] Nordfjærn T. Jørgensen S. Rundmo T. (2012). Cultural and socio-demographic predictors of car accident involvement in Norway, Ghana, Tanzania and Uganda. Saf. Sci. 50, 1862–1872. doi: 10.1016/j.ssci.2012.05.003

[ref39] O’Farrell D. L. Baynes K.-L. M. Pontes H. D. Griffiths M. Stavropoulos V. (2020). Depression and disordered gaming: does culture matter? Int. J. Ment. Heal. Addict., 1–9. doi: 10.1007/s11469-020-00231-1

[ref40] Oei T. P. S. Sawang S. Goh Y. W. Mukhtar F. (2013). Using the depression anxiety stress scale 21 (DASS-21) Across cultures. Int. J. Psychol. 48, 1018–1029. doi: 10.1080/00207594.2012.755535, PMID: 23425257

[ref41] Office of the Surgeon General (US), Center for Mental Health Services (US) National Institute of Mental Health (US) (2001). “Culture Counts: The Influence of Culture and Society on Mental Health,” in Mental Health: Culture R, and Ethnicity: A Supplement to Mental Health: A Report of the Surgeon General. Rockville (MD): Substance Abuse and Mental Health Services Administration (US) (United States: Department of Health and Human Services, U. S. Public Health Service). 20669516

[ref42] Olsson U. H. Foss T. Troye S. V. Howell R. D. (2000). The performance of ML, GLS, and WLS estimation in structural equation modeling Under conditions of misspecification and nonnormality. Struct. Equ. Model. Multidiscip. J. 7, 557–595. doi: 10.1207/S15328007SEM0704_3

[ref43] Osman A. Wong J. L. Bagge C. L. Freedenthal S. Gutierrez P. M. Lozano G. (2012). The depression anxiety stress Scales-21 (DASS-21): further examination of dimensions, scale reliability, and correlates. J. Clin. Psychol. 68, 1322–1338. doi: 10.1002/jclp.21908, PMID: 22930477

[ref44] Pontes H. M. Griffiths M. D. (2015). Measuring DSM-5 internet gaming disorder: development and validation of a short psychometric scale. Comput. Hum. Behav. 45, 137–143. doi: 10.1016/j.chb.2014.12.006

[ref45] Pontes H. M. Stavropoulos V. Griffiths M. D. (2017). Measurement invariance of the internet gaming disorder scale-short-form (IGDS9-SF) between the United States of America, India and the United Kingdom. Psychiatry Res. 257, 472–478. doi: 10.1016/j.psychres.2017.08.013, PMID: 28837939

[ref46] Scholten S. Velten J. Bieda A. Zhang X. C. Margraf J. (2017). Testing measurement invariance of the depression, anxiety, and stress scales (DASS-21) across four countries. Psychol. Assess. 29, 1376–1390. doi: 10.1037/pas0000440, PMID: 28125249

[ref47] Seymour K. E. Chronis-Tuscano A. Iwamoto D. K. Kurdziel G. MacPherson L. (2014). Emotion regulation mediates the association Between ADHD and depressive symptoms in a community sample of youth. J. Abnorm. Child Psychol. 42, 611–621. doi: 10.1007/s10802-013-9799-8, PMID: 24221724 PMC4207628

[ref48] Shekriladze I. Javakhishvili N. Chkhaidze N. (2021). Culture related factors may shape coping During pandemics. Front. Psychol. 12:634078. doi: 10.3389/fpsyg.2021.634078, PMID: 34093315 PMC8170015

[ref49] Shrout P. E. Yager T. J. (1989). Reliability and validity of screening scales: effect of reducing scale length. J. Clin. Epidemiol. 42, 69–78. doi: 10.1016/0895-4356(89)90027-9, PMID: 2913189

[ref50] Silva H. A. Passos M. H. Oliveira V. M. Palmeira A. C. Pitangui A. C. Araújo R. C. (2016). Short version of the Depression Anxiety Stress Scale-21: is it valid for Brazilian adolescents? Einstein 14, 486–493. doi: 10.1590/s1679-45082016ao3732, PMID: 28076595 PMC5221374

[ref51] Sinclair S. J. Siefert C. J. Slavin-Mulford J. M. Stein M. B. Renna M. Blais M. A. (2012). Psychometric evaluation and normative data for the depression, anxiety, and stress scales-21 (DASS-21) in a nonclinical sample of U.S. adults. Eval. Health Prof. 35, 259–279. doi: 10.1177/0163278711424282, PMID: 22008979

[ref52] Sweetland A. C. Belkin G. S. Verdeli H. (2014). Measuring depression and anxiety in sub-saharan Africa. Depress. Anxiety 31, 223–232. doi: 10.1002/da.22142, PMID: 23780834 PMC4109689

[ref53] Teo Y. C. Hj Yusuf A. Alice Lim W. P. Ghazali N. B. Abd Rahman H. Lin N.. (2019). Validation of DASS-21 among nursing and midwifery students in Brunei. J. Public Health 27, 387–391. doi: 10.1007/s10389-018-0947-z

[ref54] ter Meulen W. G. Draisma S. van Hemert A. M. Schoevers R. A. Kupka R. W. Beekman A. T. F.. (2021). Depressive and anxiety disorders in concert–A synthesis of findings on comorbidity in the NESDA study. J. Affect. Disord. 284, 85–97. doi: 10.1016/j.jad.2021.02.004, PMID: 33588240

[ref55] Tonsing K. N. (2014). Psychometric properties and validation of Nepali version of the depression anxiety stress scales (DASS-21). Asian J. Psychiatr. 8, 63–66. doi: 10.1016/j.ajp.2013.11.001, PMID: 24655630

[ref56] Triandis H. C. Gelfand M. J. (1998). Converging measurement of horizontal and vertical individualism and collectivism. J. Pers. Soc. Psychol. 74, 118–128. doi: 10.1037/0022-3514.74.1.118

[ref57] Vahedian-Azimi A. Moayed M. S. Rahimibashar F. Shojaei S. Ashtari S. Pourhoseingholi M. A. (2020). Comparison of the severity of psychological distress among four groups of an Iranian population regarding COVID-19 pandemic. BMC Psychiatry 20:402. doi: 10.1186/s12888-020-02804-9, PMID: 32770975 PMC7414274

[ref58] Vignola R. C. Tucci A. M. (2014). Adaptation and validation of the depression, anxiety and stress scale (DASS) to Brazilian Portuguese. J. Affect. Disord. 155, 104–109. doi: 10.1016/j.jad.2013.10.031, PMID: 24238871

[ref59] Wang K. Shi H. S. Geng F. L. Zou L. Q. Tan S. P. Wang Y.. (2016). Cross-cultural validation of the depression anxiety stress Scale-21 in China. Psychol. Assess. 28, e88–e100. doi: 10.1037/pas0000207, PMID: 26619091

[ref60] Wireko-Gyebi S. (2020). Data for: COVID-19 induced DAS. Mendeley Data 12:5464. doi: 10.17632/xz85fhtm6f.1

[ref61] Yıldırım A. Boysan M. Kefeli M. C. (2018). Psychometric properties of the Turkish version of the depression anxiety stress Scale-21 (DASS-21). British J. Guid. Counsel. 46, 582–595. doi: 10.1080/03069885.2018.1442558

[ref62] Zanon C. Brenner R. E. Baptista M. N. Vogel D. L. Rubin M. Al-Darmaki F. R.. (2020). Examining the dimensionality, reliability, and invariance of the depression, anxiety, and stress Scale-21 (DASS-21) Across eight countries. Assessment 28, 1531–1544. doi: 10.1177/1073191119887449, PMID: 31916468

[ref63] Zhang J. Lu H. Zeng H. Zhang S. Du Q. Jiang T.. (2020). The differential psychological distress of populations affected by the COVID-19 pandemic. Brain Behav. Immun. 87, 49–50. doi: 10.1016/j.bbi.2020.04.031, PMID: 32304883 PMC7156946

[ref64] Zhang M. Zhang J. Zhang F. Zhang L. Feng D. (2018). Prevalence of psychological distress and the effects of resilience and perceived social support among Chinese college students: does gender make a difference? Psychiatry Res. 267, 409–413. doi: 10.1016/j.psychres.2018.06.038, PMID: 29960938

