# Supplementary material for: The Depression Anxiety Stress Scale 8-Items Expresses Robust Psychometric Properties as an Ideal Shorter Version of the Depression Anxiety Stress Scale 21 Among Healthy Respondents From Three Continents
Source: Front Psychol. 2022 Mar 24;13:799769. doi: 10.3389/fpsyg.2022.799769 (PMC9044488; doi:10.3389/fpsyg.2022.799769)
Supplement: Supplementary file 1 [file Table_1.docx]

Supplementary Table 1. Invariance of factor structures of the Depression Anxiety Stress Scale 8 (DASS-8) and DASS-12 across gender groups in the international sample

| **Model** | **Invariance levels** | **χ^2^** | **df** | ***p*** | **Δχ^2^** | **Δdf** | ***p*(Δχ^2^)** | **CFI** | **ΔCFI** | **TLI** | **ΔTLI** | **RMSEA** | **ΔRMSEA** | **SRMR** |
| --- | --- | --- | --- | --- | --- | --- | --- | --- | --- | --- | --- | --- | --- | --- |
| DASS-8 | Males  Females  Configural  Metric  Strong  Strict | 41.77  41.84  83.60  90.31  99.20  170.69 | 17  17  34  39  45  53 | 0.001  0.001  0.001  0.001  0.001  0.001 | 6.71  8.89  71.49 | 5  6  8 | 0.243  0.180  0.001 | 0.99  0.99  0.99  0.99  0.99  0.98 | 0.00  0.00  0.01 | 0.98  0.99  0.98  0.99  0.99  0.97 | -0.01  0.00  0.02 | 00.05  0.04  0.03  0.03  0.03  0.04 | 0.00  0.00  -0.01 | 0.02  0.02  0.02  0.02  0.03  0.04 |
| Korean DASS-12 | Males  Females  Configural  Metric  Strong  Strict | 115.75  178.68  294.43  307.20  313.79  429.21 | 51  51  102  111  117  129 | 0.001  0.001  0.001  0.001  0.001  0.001 | 12.77  6.59  115.43 | 9  6  12 | 0.173  0.360  0.001 | 0.97  0.97  0.97  0.97  0.97  0.95 | 0.00  0.00  0.02 | 0.96  0.96  0.96  0.96  0.96  0.95 | 0.00  0.00  0.01 | 0.05  0.06  0.04  0.04  0.03  0.04 | 0.00  0.00  -0.01 | 0.04  0.03  0.03  0.03  0.04  0.05 |

*χ*^2^: chi-square; df: degrees of freedom; CFI: comparative fit index; TLI: Tucker–Lewis index; RMSEA: root mean square error of approximation; CI: confidence interval; SRMR: standardized root mean residual.
