# Supplementary material for: The Depression Anxiety Stress Scale 8-Items Expresses Robust Psychometric Properties as an Ideal Shorter Version of the Depression Anxiety Stress Scale 21 Among Healthy Respondents From Three Continents
Source: Front Psychol. 2022 Mar 24;13:799769. doi: 10.3389/fpsyg.2022.799769 (PMC9044488; doi:10.3389/fpsyg.2022.799769)
Supplement: Supplementary file 2 [file Table_2.docx]

**Supplementary Table 2.** Invariance of factor structures of the Depression Anxiety Stress Scale 8 (DASS-8) and DASS-12 across groups of English-speaking students and other forms of employment

| **Model** | **Invariance levels** | **χ^2^** | **df** | ***P*** | **Δχ^2^** | **Δdf** | ***p*(Δχ^2^)** | **CFI** | **ΔCFI** | **TLI** | **ΔTLI** | **RMSEA** | **ΔRMSEA** | **SRMR** |
| --- | --- | --- | --- | --- | --- | --- | --- | --- | --- | --- | --- | --- | --- | --- |
| DASS-8 | Students  Other employment  Configural  Metric  Strong  Strict | 30.38  69.77  145.13  155.01  161.53  176.39 | 17  17  34  39  45  53 | 0.021  0.001  0.001  0.001  0.001  0.001 | 9.79  6.52  14.86 | 5  6  8 | 0.079  0.367  0.062 | 0.99  0.97  0.96  0.96  0.96  0.95 | 0.00  0.00  0.01 | 0.98  0.95  0.93  0.94  0.94  0.95 | -0.01  0.00  -0.01 | 0.05  0.07  0.06  0.05  0.05  0.05 | 0.01  0.00  0.00 | 0.03  0.03  0.05  0.06  0.07  0.07 |
| Korean DASS-12 | Students  Other employment  Configural  Metric  Strong  Strict | 91.55  213.30  413.36  427.76  441.50  469.42 | 51  51  102  111  117  129 | 0.036  0.001  0.001  0.001  0.001  0.001 | 94.98  152.77  442.47 | 9  6  12 | 0.106  0.033  0.006 | 0.97  0.93  0.91  0.91  0.91  0.91 | 0.00  0.00  0.00 | 0.96  0.91  0.89  0.90  0.90  0.90 | -0.01  0.00  0.00 | 0.05  0.07  0.05  0.05  0.05  0.05 | 0.00  0.00  0.00 | 0.04  0.05  .06  .07  .08  .07 |

*χ*^2^: chi-square; df: degrees of freedom; CFI: comparative fit index; TLI: Tucker–Lewis index; RMSEA: root mean square error of approximation; CI: confidence interval; SRMR: standardized root mean residual.
